# Modeling Clustered DNA Damage by Ionizing Radiation Using Multinomial Damage Probabilities and Energy Imparted Spectra

**DOI:** 10.3390/ijms252312532

**Published:** 2024-11-22

**Authors:** Francis A. Cucinotta

**Affiliations:** Department of Health Physics and Diagnostic Sciences, University of Nevada Las Vegas, 4505 S. Maryland Parkway, Box 453037, Las Vegas, NV 89154, USA; francis.cucinotta@unlv.edu

**Keywords:** DNA damage, clustered double-strand breaks, ionizing radiation, high LET radiation

## Abstract

Simple and complex clustered DNA damage represent the critical initial damage caused by radiation. In this paper, a multinomial probability model of clustered damage is developed with probabilities dependent on the energy imparted to DNA and surrounding water molecules. The model consists of four probabilities: (A) direct damage of sugar-phosphate moieties leading to SSB, (B) OH^−^ radical formation with subsequent SSB and BD formation, (C) direct damage to DNA bases, and (D) energy imparted to histone proteins and other molecules in a volume not leading to SSB or BD. These probabilities are augmented by introducing probabilities for the relative location of SSB using a ≤10 bp criteria for a double-strand break (DSB) and for the possible success of a radical attack that leads to SSB or BD. Model predictions for electrons, ^4^He, and ^12^C ions are compared to the experimental data and show good agreement. Thus, the developed model allows an accurate and rapid computational method to predict simple and complex clustered DNA damage as a function of radiation quality and to explore the resulting challenges to DNA repair.

## 1. Introduction

Ionizing radiation (IR) produces, through direct and indirect action, many types of DNA lesions, such as single-strand breaks (SSBs), double-strand breaks (DSBs), and a variety of base modifications (base damage (BD)). Clustered DNA damage sites are defined as two or more elemental lesions that are formed within one or two helical turns of DNA (~base pairs) by a single radiation track [1,2,3]. Complex clustered damage is defined by the occurrence of three or more SSBs or BDs within 10 bp. IR is an efficient inducer of both complex DSB and non-DSB end structures, including the presence of BD or SSBs near DSBs and complex SSBs. The co-location of BD near DSBs or SSBs may interfere with the repair pathway choice and efficient repair [4,5,6,7,8]. Therefore, complex clustered lesions are expected to play a major role in determining the repairability of DNA lesions [4,5,6,7,8], with a wide range of implications for describing radiation-induced cell death and mutations, including chromosomal aberrations, genomic instability, and aberrant signaling pathways. The understanding of complex clustered DNA damage thus plays an important role in mechanistic models of low-dose risk assessments and in radiation oncology.

Monte-Carlo (MC) track-structure simulations of DNA damage, including simple and complex breaks, have been developed using detailed volume or atomistic models of DNA and the hydration shell surrounding DNA, including models of the early chemistry leading to indirect DNA damage [9,10,11,12,13,14,15]. MC-based simulations have made comparisons of the total yields of SSBs and DSBs to experimental data for a variety of particle types as a function of linear energy transfer (LET) while providing predictions of complex clustered damage of a variety of SSB and DSB types with or without additional base damage. Predictions of the role of BD, such as abasic sites, have been more limited in MC simulations.

Experimental approaches to describe DNA damage include pulsed-field electrophoresis (PFGE) on DSB yields, including studies with restriction enzymes [5,6,16,17,18,19,20] to study the role of damaged bases and abasic sites near SSBs or DSBs and their possible role in inhibiting repair. The use of atomic force microscopy has recently provided data on a wider variety of clustered damage types [21]. Immunohistochemistry is used to observe DNA repair foci for proteins specific to non-homologous end-joining (NHEJ), homologous recombination (HR), and other signaling pathways [22,23,24,25,26]. The kinetics of the loss of foci with time after exposure, studied with different doses, radiation qualities, and time after irradiation, has been used as an indicator of complex damage. However, experimental methods to measure the wide spectrum of damage types predicted by computational models have not been developed.

In Charlton et al. [9], MC track-structure simulations using a cylindrical volume representing a segment of DNA with 54 base pairs were used to consider the types of complex breaks induced by electrons and high LET α-particles. The results suggested a model where the spectrum of energy imparted in the volume containing DNA folded with the probability of producing DNA damage was predictive of yields for a variety of combinations of simple and complex SSBs and DSBs. In this approach, the yield of a specific damage type, *j*, per Gy, is evaluated as:

(1) $$Yield_{j}(E_{0})=c\int d\varepsilon \frac{dF(E_{0},\varepsilon )}{d\varepsilon }P_{j}(\varepsilon )$$

where *E*_0_ is the incident energy, *c* is the conversion constant for evaluating the yields as per Gy per bp (or similarly for per Gy per Dalton or per Gy per cell), and *dF/dε* is the differential distribution of the energy imparted, ε per Gy. The function $P_{j}(\varepsilon )$ is the probability of producing a specific damage type *j* for energy imparted ε. Based on track-structure MC simulations, Charlton et al. [9] found these probabilities to be largely independent of radiation quality, and a strong correlation occurs between the energy imparted to a volume model of DNA containing 54 bp and the probability of simple and complex break types. Charlton et al. [9] considered only the direct effects for SSB and DSB formation for a 54 bp segment while ignoring the BD and indirect effects and found a negligible probability of no damage above ~100 eV.

In this paper, I develop a multinomial probability model that predicts the spectrum of DNA damage types, including the yield of simple and complex DNA breaks and BD that can be applied to all types of radiation, including photons, electrons, protons, helium ions, and heavy ions. The model is based on probabilities for SSBs, DSBs, BD, and their combinations using a multinomial probability formalism. Charlton et al. [9] found that a 54 bp segment was sufficient to describe DNA damage for high LET alpha particles. However, to describe ^12^C and other heavy ions, I use a larger segment. For comparisons to the experiment, I apply the frequency distribution of energy imparted for a 5 × 5 nm cylindrical volume representing a significant fraction of a nucleosome containing ~73 bp. The model provides predictions of SSBs and DSBs of increasing complexity along with the frequency of breaks with or without BD.

## 2. Multinomial Probability Model

A multinomial distribution is a generalization of a binomial distribution extended to the case of multiple event outcomes. In applying this model to predict clustered DNA lesions, I consider four types of events that result from energy imparted to the volume: (A) direct ionization of sugar-phosphate moieties, with probability *P_A_* leading to a SSB; (B) ionization of water leading to OH^−^ radicals, with probability *P_B_*; (C) direct damage to DNA bases with probability *P_C_*; and (D) energy imparted to histone proteins and other co-located molecules in the volume not leading to SSBs or BDs, *P_D_*. DNA-protein crosslinks are not considered. Evaluating the distributions in a number of the A, B, C, and D probabilities to a high order allows for predictions of clustered damages of increasing complex clustered damage.

The threshold energy imparted for each type of damage (A, B, C, and D) varies to some extent with the threshold energy for OH^−^ production of 13 eV, and several MC-based simulation results use 17.5 eV for SSBs [10,11,12]. Threshold energies for BD ionization are reported in a similar range [27]. In order to simplify the formalism, I assume the threshold is approximately the same for each type and use a normal distribution with a central estimate of ε*_th_* = 17.5 eV and a standard deviation of 5 eV in the calculations. The assumption of a single threshold for each type of event can be removed, as discussed below.

Above the energy threshold for a single event (denoted as first order), the following condition occurs for the summed probability of each outcome,

(2) $$P_{A}+P_{B}+P_{C}+P_{D}=1$$

Because the energy thresholds for ionization across the molecules considered have similar values, the probabilities in Equation (2) are estimated simply by the fractional molecular weight of each component. As the energy imparted ε increases, the number of possible events increases. I introduce an index *J_TOT_*(*ε*) to evaluate the total number of events for a given energy imparted ε, which is found as

(3) $$J_{TOT}(\varepsilon )=Integer(\frac{\varepsilon }{\varepsilon_{th}})$$

The number of each type of event that occurs is constrained by

(4) $$J_{A}(\varepsilon )+J_{B}(\varepsilon )+J_{C}(\varepsilon )+J_{D}(\varepsilon )=J_{TOT}(\varepsilon )$$

The multinomial probability for various combinations of events is

(5) $$P(J_{A},J_{B},J_{C},J_{D},\varepsilon )=\frac{J_{TOT}(\varepsilon )!}{J_{A}!J_{B}!J_{C}!J_{D}!}P_{A}^{J_{A}}P_{B}^{J_{B}}P_{C}^{J_{C}}P_{D}^{J_{D}}$$

The probabilities of Equation (5) are enumerated, and marginal distributions are formed to evaluate various types and combinations of DNA damage. Note that, based on calculations of frequency distributions for a 5 × 5 nm cylindrical volume considered [28,29,30], the order of importance is up to *J_TOT_*~6 for low LET radiation and much higher values (*J_TOT_* > 10) of importance for high LET radiation.

As *J_TOT_* increases, complex clustered damage occurs, including multiple SSBs, DSBs, and BD within 10 bp. An application of the model will show that a large fraction of SSBs and DSBs are formed in combination with the BD for *J_TOT_* > 2. The frequency of simple SSBs is denoted as *n_SSB_*(*S*), and complex SSBs are defined as the occurrence of two SSBs on the same strand within 10 bp, denoted as *n_SSB_*(*+*). If the two SSBs are on opposite strands within 10 bp, a simple DSB occurs, denoted as *n_DSB_*(*S*). If more than one isolated SSB occurs, its frequency is denoted as *n_SSB_*(*Sm*), where *m* is the number of ‘isolated’ simple SSBs. Similarly, complex DSBs, with the frequency denoted as *n_DSB_*(*+*)*,* are the occurrence of a DSB with an additional SSB within 10 bp. More complex SSBs and DSBs containing >3 SSBs or >2 DSBs are grouped together and are denoted *n_DSB_*(*++*) and *n_SSB_*(*++*), respectively. In this report, BD is considered using the notation *n_BD_*(*m*). Analyses of the probabilities for SSB and DSB frequencies that consider the spatial distance to BD with a higher resolution than within the 73 bp segment will be considered in a future report.

Additional probabilities are needed to evaluate SSBs, DSBs, and BD and their combination probabilities. The first is to account for the spatial location of multiple SSBs in accordance with two or more within the bp ≤ 10 criteria for a DSB or a complex SSB. I assume this possibility is equally probable with an operation that adds a SSB on opposite and identical strands with the mathematical operators ${\hat{q}}_{L}$ and ${\hat{q}}_{R}$ with the magnitude $\left|{\hat{q}}_{L}+{\hat{q}}_{R}\right|=2q_{1}$. This leads to the following condition, with ${\hat{q}}_{0}$ being the mathematical operator for the introduction of an additional SSB that is farther than 10 bp apart from the previous one. The overall magnitude of these operations obeys unit probability:

(6) $$q_{0}+2q_{1}=1$$

The values in Equation (6) are dependent on the number of SSBs induced because as the number increases, they are more likely to fall within a 10 bp separation. Estimates of the probability of not producing a cluster for the addition on each additional SSB are added, and *q*_0_ were made for 73 bp segments using Monte-Carlo sampling for *J* = 2, 3, 4, 5, 6, and 7 and were found to be 0.87, 0.74, 0.6, 0.475, 0.35, and 0.12, respectively. A similar consideration holds as *J_B_* is increased, allowing for increased radical production, such that additional SSBs are added into a lesion.

A second probability is needed to estimate if an SSB or a BD is formed by an OH^−^ radical attack, with the probabilities denoted as *r*_1_ and *r*_2_, respectively. Schoel et al. [31] made an estimate of the interactions by OH^−^ radicals being 80% with bases and 20% with sugar-phosphate moieties. This estimate is combined with an estimated 65% probability of conversion to SSBs in MC codes [10,11,12] after fitting the experimental data, which leads to an overall 13% probability for SSBs caused by OH^−^ radicals. The same criteria are used here to estimate a probability for BD, which is 0.8 × 65% = 52%. This leads to the parameter estimate of *r*_1_~0.13 for conversion to a SSB and *r*_2_~0.52 for conversion to BD. Therefore *r*_3_ = *1* − *r*_1_ − *r*_2_ represents the probability that no SSB or BD were formed after OH^−^ induction.

To evaluate the terms with multiplicative probabilities, such as *P_A_P_A_*, I treat the probabilities using a mathematical operator ${\overset{⌢}{O}}_{j}$ with a numerical value, denoted by lower-case *p_j_* multiplied by the operator that combines multiple damages in the volume, considering the ≤10 bp criteria to determine the type of damage that occurs. The operator describes the lesion’s location and its possible complexity as SSBs are added into a volume representing a small DNA segment. The A-operator is defined as:

(7a) $${\overset{⌢}{A}}_{1}=p_{A}({\hat{q}}_{0}+{\hat{q}}_{L}+{\hat{q}}_{R})n_{SSB}$$

(7b) $${\overset{⌢}{A}}_{1}\cdot [1]=p_{A}n_{SSB}(S)$$

where the operand appears in square brackets. In addition, to simplify notation, the magnitude of product terms to order *J_A_* is written as

$$A_{J}=\prod_{j=1}^{J_{A}}P_{A}.$$

In the following, the *p_j_* constant factors are not shown to simplify the formula, while their values for various permutations in Equation (5) are easily identified. The second-order term *P_A_P_A_* is found to contain three branching probabilities that are weighted with the identical multinomial probabilities defined in Equation (5):

(8) $$A_{2}={\overset{⌢}{A}}_{1}[P_{1}]\to \left\{\begin{array}{c} q_{0}n_{SSB}(S2)\\ q_{1}n_{DSB}(S)\\ q_{1}n_{SSB}(+) \end{array}\right\}$$

The third-order term leads to five branches:

(9) $$A_{3}={\overset{⌢}{A}}_{1}[P_{1}P_{1}]\to \left\{\begin{array}{c} q_{0}^{2}n_{SSB}(S3)\\ 2q_{0}q_{1}n_{DSB}(S)n_{SSB}(S)\\ 2q_{0}q_{1}n_{SSB}(+)n_{SSB}(S)\\ 3q_{1}^{2}n_{DSB}(+)\\ q_{1}^{2}n_{SSB}(++) \end{array}\right\}$$

In Equation (9), *n_SSB_*(*++*) denotes the occurrence of three SSBs within 10 bp, located on a single strand. Equation (9) shows that the probability of the lesion DSB+ exceeds that of the SSB++ probability by 3-fold in their first occurrence of the third-order term, while DSB and SSB+ have equivalent weighing at both the second and third orders. Table 1 illustrates the action of the A-operator on various operands (several SSB and DSB types). A factor of 2 occurs in Equation (9) when the A-operator acts on prior lesions with two SSBs since the operator acts on both SSBs; note that a factor of 2 is also needed to obey the conservation rule of Equation (6).

The operator for the formation of SSBs and BD by OH^−^ radical attack is defined as:

(10) $${\overset{⌢}{B}}_{1}=r_{1}({\hat{q}}_{0}+{\hat{q}}_{L}+{\hat{q}}_{R})n_{SSB}+r_{2}n_{BD}(1)+r_{3}$$

Then,

(11) $$B_{1}=P_{B}=r_{1}n_{SSB}(S)+r_{2}n_{BD}(1)$$

At the first order, the *r*_3_ probability does not produce any effect; however, in general, the *r*_3_ component of the *B*-operator on any operand [*O*] is simply *r*_3_*O*.

At the second order, the first term in Equation (10) introduces clustered SSBs, and six branching probabilities occur, as follows:

(12) $$B_{2}={\overset{⌢}{B}}_{1}[P_{B}]\to \left\{\begin{array}{c} r_{1}^{2}q_{0}n_{SSB}(S2)\\ r_{1}^{2}q_{1}n_{DSB}(S)\\ r_{1}^{2}q_{1}n_{SSB}(+)\\ 2r_{1}r_{2}n_{SSB}(S)n_{BD}(1)\\ \begin{array}{l} r_{2}^{2}n_{BD}(2)\\ r_{3}B_{1} \end{array} \end{array}\right\}$$

In Equation (12), the last branch involving *B*_1_ is applied with the multinomial coefficient of Equation (5) for the *B*_2_ probability.

### 2.1. Higher-Order Terms

The higher-order terms in *A_J_* and *B_J_* or their products will have many components. For higher-order terms in *B_j_*, an accurate approximation is to keep only the terms up to *r*_1_^2^ since for higher powers, *p* > 2, *r*_1_*^p^* << 1. For the case of a mixture of three and higher-order terms with the involvement of OH^−^ radicals, this leads to the approximation:

(13) $$B_{J}=\prod_{j=1}^{J_{B}}P_{B}\approx B_{2}{\left[r_{2}n_{BD}(J_{B}-2)+r_{3}\right]}^{J_{B}-2},\begin{array}{cc} & J_{B}>2 \end{array}$$

Useful recursion relations for the B-operator acting on products of A-terms are found as:

(14) $${\overset{⌢}{B}}_{1}[A_{J_{A}}]=r_{1}A_{J_{A}+1}+[r_{2}n_{BD}(1)+r_{3}]A_{J_{A}}$$

And for *B*_2_ acting on A-terms:

(15) $${\overset{⌢}{B}}_{1}[B_{1}A_{J_{A}}]=r_{1}^{2}A_{J_{A}+2}+2r_{1}[r_{2}n_{BD}(1)+r_{3}]A_{J_{A}+1}+[r_{2}^{2}n_{BD}(2)+2r_{2}r_{3}n_{BD}(1)+r_{3}^{2}]A_{J_{A}}$$

In Equations (14) and (15) and several equations below, for compactness of notation, the terms are written with additions; however, each term in these equations represents a probability of a specific damage cluster event.

Higher-order terms in the *C*, *D*, or mixtures of *C* and *D* probabilities are evaluated as simple products. The terms with *B* or *A* probabilities with *C* and *D* to any order are evaluated as simple products; however, the *A* and *B* terms are more complex to evaluate. Mixtures with *B* probabilities then involve the use of the approximation of Equation (13) and recursion relations of Equations (14) and (15). For mixtures of heterogeneous terms, the order of the probabilities is invariant. The results for first- and second-order terms *J_TOT_*-*J_D_* are shown in Table 2, and in Table 3 for the third order in *J_TOT_*-*J_D_*. For *J_TOT_*-*J_D_* = 4, there are 15 terms amongst *A*, *B*, and *C,* with several of the fourth-order terms cumbersome to evaluate because of the many components (Table 4).

The terms fifth order and higher order in the *A* probability become increasingly difficult to evaluate. However, at large values of energy imparted, the dominance of complex clustered DNA damage is expected. This observation leads to an approximation method to evaluate these probabilities. First, I note that terms to order *J_A_* must follow an inherent binomial probability rule for the factor ${\left(q_{0}+2q_{1}\right)}^{J_{A}-1}$. Therefore, the expansion in terms of increasing powers of *q*_0_(*2q*_1_) should be the basis for evaluating higher terms. This expansion is described using the binomial coefficients:

(16) $${\left(q_{0}+2q_{1}\right)}^{J}=\sum_{k=0}^{J}\left(\begin{array}{c} J\\ k \end{array}\right)q_{0}^{J-k}{\left(2q_{1}\right)}^{k}$$

This form occurs in Equation (9) for *A*_3_ and the fourth-order term in the *A*-probability, which is found as:

(17) $$A_{4}=T_{1}+T_{2}+T_{3}+T_{4}$$

with

$$\begin{array}{l} T_{1}=q_{0}^{3}n_{SSB}(S4)\\ T_{2}=3q_{0}^{2}q_{1}[n_{DSB}(S)n_{SSB}(S2)+n_{SSB}(+)n_{SSB}(S2)]\\ T_{3}=q_{0}q_{1}^{2}\begin{array}{c} \left[6n_{DSB}(+)n_{SSB}(S)+2n_{SSB}(++)n_{SSB}(S)+2n_{DSB}(S)n_{SSB}(+\right)\\ +n_{DSB}(+)n_{DSB}(S)+n_{SSB}(+)n_{SSB}(+)] \end{array}\\ T_{4}=q_{1}^{3}[7n_{DSB}(++)+n_{SSB}(+++)] \end{array}$$

As noted above, the value of *q*_0_ decreases as *J_A_* increases. Therefore, the fifth-order and higher-order terms are dominated by SSBs and DSBs of increasing complexity and are limited to complex DSB++ or larger lesions for *J_A_* >> 1. It follows that for *J_A_* > 4, an accurate approximation is to evaluate contributions in powers up to the third order in *q*_1_ (i.e., up to *q*_1_^3^) and tally all the higher-order terms in the binomial expansion into the *n_DSB_*(*++*) and *n_SSB_*(++) categories using the binomial expansion coefficients (Equation (16)) with terms similar to Equation (17). This summation is quite transparent when one notes that the summation of the binomial coefficients is given by 2^J^, while *q*_1_ is limited to ½ at large *J_A_*. Therefore, for *J_A_* >> 1 where *q_0_*~0, the summation of the series limits to an effective population of highly complex DSBs or SSBs. To facilitate the calculation of higher-order terms in *J_A_*, the following is useful:

(18) $$A_{J}=q_{0}n_{SSB}(S)A_{J-1}+({\overset{⌢}{q}}_{L}+{\overset{⌢}{q}}_{R})n_{SSB}(S)A_{J-1}$$

With the approximation that the cubic terms in *q*_1_ (*q*_1_^3^) and higher powers are counted in the *n_DSB_*(*++*) population.

### 2.2. Summations of Probabilities for Simple and Complex Damage Probabilities

Total yields for SSBs, DSBs, and BD or mixtures are found using marginal distributions formed by summing various probabilities where at least a lesion type of interest occurs or other criteria. For example, the probability for one or more DSBs is:

(19) $$P(DSB\geq 1)=\sum_{J_{A}>1,J_{B}>1}^{J_{TOT}}\frac{J_{TOT}(\varepsilon )!}{J_{A}!J_{B}!J_{C}!J_{D}!}\bar{P_{A}^{J_{A}}P_{B}^{J_{B}}}P_{C}^{J_{C}}P_{D}^{J_{D}}$$

where $\bar{P_{A}^{n_{A}}P_{B}^{n_{B}}}$ indicates only to include combinations of the A and B probabilities where a DSB occurs.

## 3. Results

**Figure 1:**
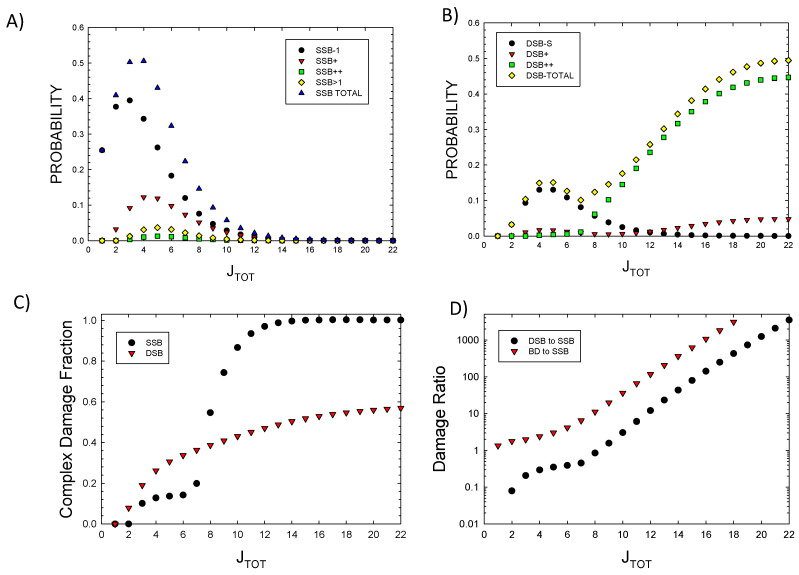
Probabilities of various types of DNA lesions versus *J_TOT_*. (**A**) Single-strand breaks, (**B**) double-strand breaks, (**C**) the fraction of complex SSBs and DSBs. (**D**) The ratios of DSBs to SSBs and BD to SSBs.

To estimate the *p_j_* probabilities, the molecular weight of each component is considered. The average molecular weight of each of the eight histone proteins is 14 kDa, and of DNA, 0.65 kDa per bp. The number of water molecules varies under specific conditions, with estimates of ~3000 per nucleosome [32]. Based on these estimates, calculations were made with approximate values of *p_A_* = 0.2, *p_B_* = 0.2, *p_C_* = 0.2, and *p_D_* = 0.4, representing estimates of the fraction of energy imparted to the 5 × 5 nm target volume by each component. Figure 1A,B show the probabilities for SSBs and DSBs of various complexity as a function of *J_TOT_*. The results show the dominance of DSB++ for *J_TOT_* > ~6. In Figure 1C, the results for the fraction of complex SSBs and DSBs versus *J_TOT_* are shown. Complex DSBs dominate with increasing *J_TOT_* due to the impacts of clustering, which reduces the probability of complex SSBs at large *J_TOT_*. Large damage clusters are more likely to form complex DSBs, as predicted by the higher-order terms described above. Isolated SSBs are found with some frequency for *J_TOT_* up to ~10, as they can occur in the 73 bp segment at some distance from a main cluster.

**Figure 2:**
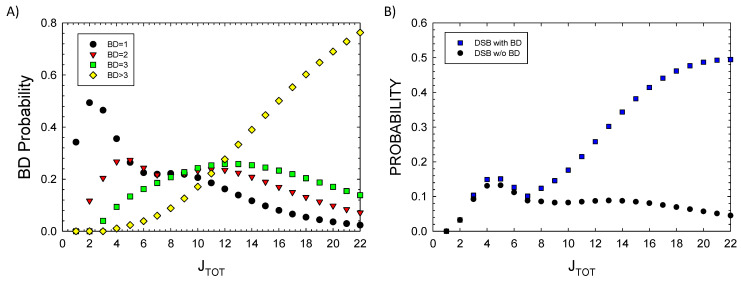
Model results for (**A**) probability of single or multiple base damage (BD) showing contributions from the number clustered BD frequencies for increasing damage numbers, J_TOT_, and (**B**) DSB probability with or without BD. Calculations assume a 73 bp segment.

In Figure 1D, the prediction of the ratio of DSBs to SSBs and BD to SSBs is shown. SSBs and BD occur with similar probability at a low *J_TOT_* (<5), while BD and DSB probabilities greatly exceed SSBs at a large *J_TOT_*. DSBs exceed SSBs due to increased clustering, leading to the dominance of a complex DSB relative to SSB at a large *J_TOT_*. Detailed considerations of BD clustering will be described in a future report. Here, preliminary observations can be made based on the results of Figure 2A, where probabilities for 1, 2, 3, or >3 BDs are plotted versus *J_TOT_*. A more detailed analysis of BD clustering and their occurrence near SSBs or DSBs is not possible at small values of *J_TOT_*; however, for a *J_TOT_* > *10,* which is important for high LET radiation, these results suggest BD will be co-located within 10 bp to SSBs or DSBs in almost all events. In Figure 2B, the probability of a DSB occurrence with and without BD formation is estimated by setting *r*_2_ = 0 in applying the multinomial probabilities for cluster formalism. As expected, very few DSBs are predicted to be formed at a large *J_TOT_* with the BD not co-located.

### Predictions for Radiation-Induced DSBs

**Figure 3:**
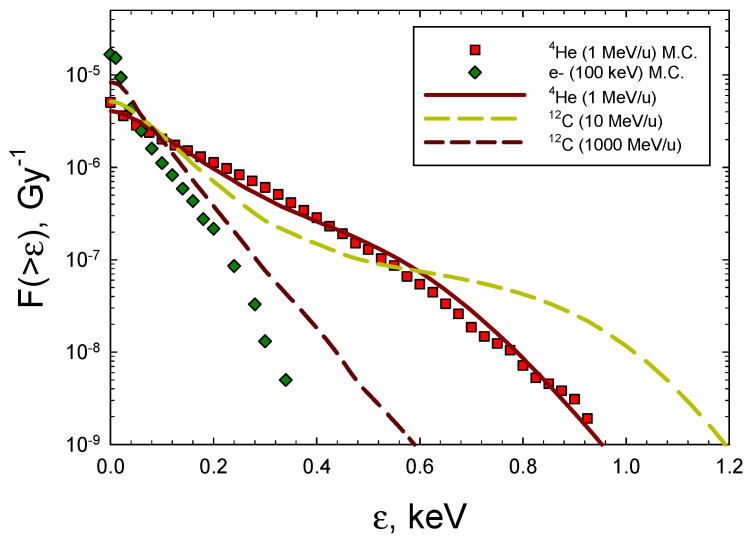
Frequency distributions of energy imparted to a 5 × 5 nm cylindrical volume for 100 keV electrons, ^4^He, and ^12^C ions. Symbols are Monte-Carlo results from Charlton et al. [28] for ^4^He and Nikjoo et al. [29] for electrons. Lines show results from calculations of the model of Cucinotta et al. [30].

Predictions for 100 keV electrons representative of X-rays and ^4^He and ^12^C ions with energies from 0.1 to 10,000 MeV/u were made and compared to the experimental data. The electron results for the frequency distributions from the Monte-Carlo results of Nikjoo et al. [29] were fitted, assuming the integral spectrum is an exponential function. For ions, we used the formalism developed by Cucinotta et al. [30], which combines the direct effects, where the ion passes through the target volume, and the δ-ray effects, where the ion passes outside of the target volume. For these δ-ray events, electron spectra as a function of radial distance from the ions’ path are folded with Monte-Carlo results for the electron energy-imparted spectra from [29]. In Figure 3, representative frequency distributions are shown for 100 keV electrons ^4^He of kinetic energy 1 MeV (LET = 104 keV/μm), ^12^C ions of kinetic energy 10 MeV/u (LET = 166 keV/μm), and ^12^C ions of kinetic energy 1000 MeV/u (LET = 8 keV/μm). The results of Figure 3 show that the analytic formalism is in good agreement with the MC simulations for low-energy ^4^He ions [28]. Table 5 shows the spectra of DNA yields for the radiation types considered in Figure 3. The integral DSB yield for 100 keV electrons of 9.9 DSB per Gy per Gbp can be compared to the values from the experiments on human skin fibroblasts or V79 cells of 6.0 for 250 kVp X-rays, 7.6 for ^60^Co gamma-rays, 11.9 for ^137^Cs gamma-rays, and for 15 MeV electrons, 6.01 [33,34,35,36], which were reported using several experimental methods. Optimization of the values of *p_i_* by fits to the experimental data was not made. However, we note that introducing relative variations of ±20% leads to similar relative changes in the predicted break yields. Yields of BD compared to the DSBs are about 10-fold higher, dependent on the radiation quality. If complex BD is considered as two or more BDs in a small DNA segment, a much higher probability of complex BD compared to complex DSBs is suggested; however, a large fraction of both these types will occur in the same lesion.

**Figure 4:**
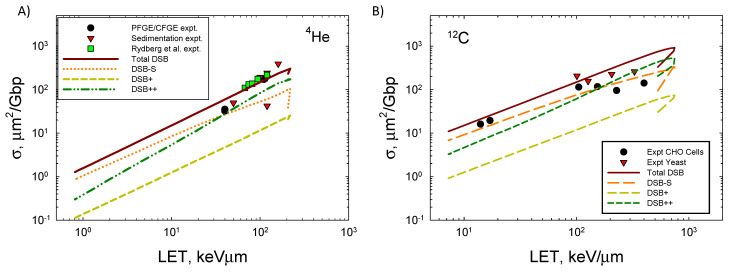
Comparison of model to experimental data [32,33,34,35,36,37] for (**A**) ^4^He ions and (**B**) ^12^C ions of action cross-section versus LET for DSBs. Calculations correspond to 40 ion energies from 0.1 MeV/u to 10,000 MeV/u.

For ions, we considered the damage frequencies expressed as an action cross-section in units of the number of breaks per Gbp per particle, which is found as:

(20) $$\sigma_{j}(E_{0})=\frac{10^{9}}{n_{BP}{\bar{z}}_{F}}\frac{LET}{6.24}\int d\varepsilon \frac{dF(E_{0},\varepsilon )}{d\varepsilon }P_{j}(\varepsilon )$$

where dF/dε is normalized to unity, *n_BP_* = 73, and ${\bar{z}}_{F}$ is the frequency mean specific energy to the volume. Figure 4 shows the LET dependence of the DSB formation for ^4^He and ^12^C ions compared to the experimental data [33,34,35,36,37,38,39]. Agreement of the model to the measurements is good, especially when the variation in data reported from different labs employing PFGE or sedimentation is considered [33]. At the highest LET values for both ions, experimental methods are expected to undercount the number of DSBs that occur. Here, experimental methods such as PFGE and sedimentation are expected to underestimate the DSB counts because more than one DSB in an extended region of DNA will be identified as a single DSB. A preliminary estimate correction for multiple DSBs within the 73 bp segment suggests a correction of ~50% at high LET if the DSB++ is counted as a single DSB. Future studies with the present model will estimate the correction considering larger regions of DNA, which will be especially important for high energy and charge (HZE) ions. Action cross-sections σ decrease at a high LET as the ion’s velocity decreases and indicates an overkill effect, which leads to a decrease in relative biological effectiveness (RBE) ~σ/LET.

**Figure 5:**
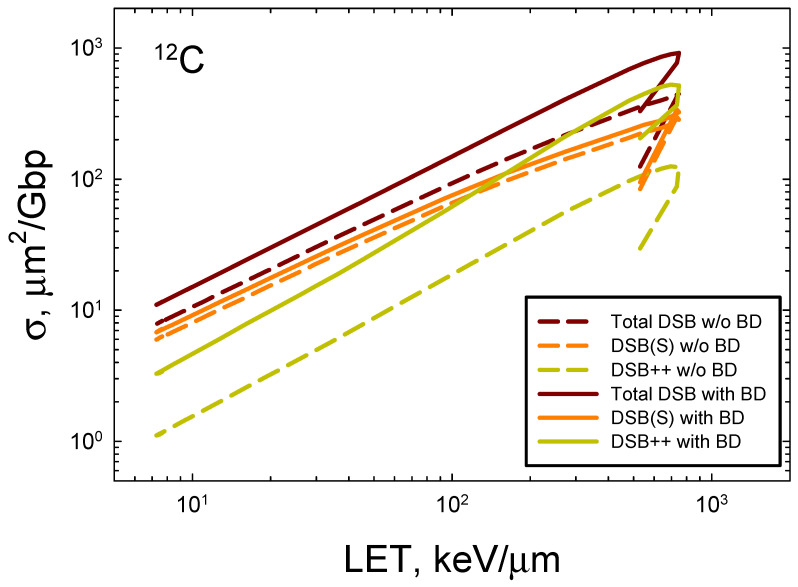
Prediction of action cross-sections for DSBs with and without (w/o) the occurrence of base damage in the 73 bp DNA structure for ^12^C ions.

In Figure 5, we consider the prediction of action cross-sections for the DSBs induced by ^12^C with and without the occurrence of base damage in the same 73 bp DNA structure using the approach described in Figure 2B. Reductions of about 2-fold occur at high LET and reductions of ~30% for relativistic ^12^C ions. The largest reduction is for DSB++ lesions, which is more than 4-fold at LET > 100 keV/μm. These results reveal the expected severe clustering that occurs for high LET ions that go beyond the contributions of clustered breaks alone.

## 4. Discussion

In this paper, a novel approach to describing clustered DNA damage using multinomial probabilities was developed. The use of energy-imparted spectra for a 5 × 5 nm cylindrical volume offers a fast-computational approach for any radiation type in comparison to the more computationally expensive application of stochastic MC-based radiation tracks to model DNA damage [9,10,11,12,13,14]. The order of averaging made using frequency distributions is a basic difference compared to full MC track-structure simulations, which average the results over many MC histories using either volume models of DNA or scoring ionizations in atomistic DNA model structures. These descriptions are often combined with kinetics models of early chemical reactions leading to indirect effects. The MC approach averages over the orientation of the track relative to the DNA structures, while simulations take many hours of CPU time on typical computer workstations and often ignore the role of BD.

The developed model uses frequency spectra that average the energy imparted over a similar volume used in MC track-structure simulations, which are then combined with the multinomial probability functions to predict DNA lesions. This results in predictions of the full DNA damage spectra obtained in a computationally efficient manner (CPU time ~1 s) for any radiation type. This aspect is highly favorable for space radiation studies where 28 elemental groups over wide energy ranges (<0.1 MeV/u to ~50 GeV/u) are typically considered [40]. Also, in hadron therapy with ^12^C or other ions, the extensive distribution of secondary particles and energies arising from Coulomb slowing down, nuclear fragmentation [41], and spallation necessitate computationally efficient models.

Measurements with the PFGE are the main source of experimental data for DSB yields; however, they are expected to underestimate the yields when multiple DSBs are produced within several 10s of kbp [33,42,43]. Therefore, a comparison to heavy ions, such as ^56^Fe, is not included in the present work. In future work, radial distributions of the energy imparted for high-charge and energy (HZE) ions [30] and models of higher-order DNA structures will be used to make comparisons to heavy ion DNA damage experiments [38,39,44].

The present approach is similar to full MC track-structure simulations in the use of a simple energy threshold for SSBs, BD, and radical formation. However, in the present calculations, a normal distribution of energy thresholds is used because it is unlikely that a single energy threshold occurs in the ionizations leading to SSBs or BD formation when one considers the complexity of the molecules involved. The use of an identical threshold in Equations (4) and (5) could be relaxed by allowing the *J_i_* indexes to increase with a more complex dependency on the energy imparted; however, this is unlikely to lead to important changes at the higher values of energy imparted where many terms contribute to damage production.

The use of cluster probabilities (*q*_0_ or *q*_1_) based on simple random probability criteria ignores the possible details of radiation tracks, such as low-energy electrons and their distinct angular trajectories versus straight-line trajectories of higher-energy electrons produced by ions. It is likely that the estimates of *q*_0_ for increasing *J_A_**/**J_B_* based on a random distribution overestimate the clustering that occurs, which is suggested by the predictions of a higher probability of DSB++ compared to the MC track-structure simulation predictions [10,11,12,13,14]. An alternative can be considered using a weighted combination of random distribution and a probability of straight-line motion of radiation tracks. We used the values of *r*_1_ and *r*_2_ related to the indirect effects of radical production on water molecules based on the MC estimates [10,11] and the estimate from Scholes et al. [31]. In future work, the values of various parameters, as fitted to experimental data, can be considered to investigate if a deviation from a random pattern or the MC model estimates are suggested.

The use of enzymatic probes, such as endonuclease III (Nth) to detect oxidized pyrimidines, formamidopyrimidine-DNA glycosylase (Fpg) to detect oxidized purines, and Nfo protein (endonuclease IV) to detect abasic sites, have revealed higher frequencies of clustered BD compared to DSBs [5,6,8,17,18,35,45]. The present model only predicts a generic category of BD. For ^137^Cs gamma-rays, Tsao et al. [35] report 9.5, 11.87, and 10.68 per Gbp per Gy for endo IV, Fpg, and endo III clusters, respectively. As seen in Table 5, 100 keV electrons show yields of 17.2, 8.7, and 4.4 per Gbp per Gy for the BD clusters of 2, 3, and >3, respectively. These values would be increased in an approximately linear fashion with increases in the value of the *r*_2_ parameter. Radiation yields for all possible base modifications have not been reported, and additional BD lesions are likely [46,47]. It would be interesting to introduce an empirical approach to model-specific base lesions for X-rays or gamma-rays to explore their ability to predict equivalent lesions for high LET radiation using the present approach. In addition, additional damages are produced in the processing of SSBs or BD in base excision repair (BER) or other pathways [5,46], and it is useful to predict the initial rates of production for comparison purposes.

A main focus of the current approach is to develop a model that can be compared to experimental data while considering the distribution of DNA end-structures that are substrates for various repair pathways for use in mathematical models of DNA repair [48,49,50]. The repair of DSBs is cell cycle-dependent, with NHEJ being dominant in the G0/G1 phases and HR in the late S phase and G2. The NHEJ pathway is error-prone, while HR is the only faithful repair pathway. The alternative end-joining pathway (alt-EJ) and single-strand annealing (SSA) are more error-prone and expected to play an increased role in complex DSB processing when NHEJ or HR are inhibited. The presence of BD near SSBs [8,51] or BD and SSBs near DSBs are possible impairments to faithful repair [4,7,8,19,51,52]. The position of the BD relative to the ligated ends, the multitude (1, 2, 3, etc.) of BD clusters, including bi-stranded clusters, along with the clustering of SSBs and DSBs, likely play impairment roles in the impairment of NHEJ or HR pathways, leading to error-prone repair, such as alt-EJ or SSA. The high frequency of complex DSBs leading to small DNA fragments is shown to reduce the efficiency of Ku70/80 from binding to DNA [53,54]. Differences in clustered DSBs, such as DSB++, between 100 keV electrons and high LET alpha particles and ^12^C ions, are only about 2-fold in the present results; however, larger differences will occur when larger DNA structures are considered, or for lesions such as DSB+++. These aspects will be considered in future work using the present approach.

The wide range of distinct DNA lesions that will occur across a cell thus points to the differences between low and high doses and low and high LET radiation. The higher frequency of BD or SSBs is likely dominant at low doses of low LET irradiation where few DSBs per cell are formed, while, as the dose is increased, the number of clustered DSBs increases, such that DSB repair likely becomes more dominant in cellular responses. For high LET radiation, clustered DSBs will occur at all doses, and the importance of the additional BD and clustered SSBs is suggested to play a smaller role. However, an exception is the large transverse distribution of the delta-rays (high energy electrons) produced about the path of HZE ions, leading to frequent low-dose cellular energy deposition in many cells not traversed by the ion [55]. This aspect should play an important role in risk assessments for the low dose and dos-rate space radiation exposures.

The range of complexity shown here that increases with ionization density (or LET) is consistent with the so-called “overkill effect” used to describe the high LET dependence on radiation effects. The increase in ionization density (or LET) presents a transition from the dominance of simple DNA damage to highly complex DSBs, as shown by the developed formalism. Saturation due to highly complex DSBs is predicted as the energy imparted increases to high levels (>~200 eV), and highly complex DSBs are predicted to dominate the initial damage [1,56]. In addition, saturation of biological action cross sections often reflects an underlying geometric damage area such as observed for cell inactivation and mutation [56,57] and a similar effect is observed for DSB yields from heavy ions [38]. The more complex damage likely favors cell death, while intermediate damage levels favor misrepair and mutation in the repair of complex SSBs and BD and single DSBs with additional BD.

## 5. Conclusions

In this paper a biophysics model for predicting yields of simple and complex clustered DNA damage induced by arbitrary radiation types was described. In this novel approach, multinomial probabilities are combined with a frequency distribution for a small DNA segment represented by a 5 × 5 nm cylindrical volume. The model calculations showed good agreement with the experimental data for DSB induction. The approach leads to an accurate, fast-computational approach with several orders of magnitude less computational expense compared to the stochastic Monte-Carlo track-structure simulations. The current paper focused on complex DSBs. Future work will extend the approach to provide more detailed descriptions of BD clustering and specific BD lesions. Parameters for radical attack leading to SSBs and BD were based on the Monte-Carlo model fits reported by others [10,11]. Future efforts will focus on considering the parameter values based on experimental data as a function of oxygen tensions. Values for parameters describing SSB clustering, *q*_0_ and *q*_1_, used here, were based on the random clustering of SSBs in the volume. An alternative model will be developed in future work to consider the balance between the random induction of SSBs in the volume and those produced along straight-line radiation track motion in the volume. Ultimately, the developed models will be used to consider clustered DNA damage processing and resulting mutations.

## Figures and Tables

**Table 1:** Action of the A-operator on several SSB and DSB operands of increasing complexity.

Operand	Branching Probabilities
SSB(S)	q_0_ n_SSB_(S2) + q_1_[n_SSB_(+)+n_DSB_(S)]
SSB(+)	q_0_ n_SSB_(+)n_SSB_(S) + q_1_[n_SSB_(++)+n_DSB_(+)]
DSB(S)	q_0_ n_DSB_(S)n_SSB_(S) + 2q_1_n_DSB_(+)
DSB(+)	q_0_ n_DSB_(+)n_SSB_(S) + 2q_1_n_DSB_(++)
DSB(S) × SSB(S)	q_0_ n_DSB_(S)n_SSB_(S2) + q_1_[n_DSB_(+)n_SSB_(S) + n_DSB_(S)n_DSB_(S)]
SSB(+) × SSB(S)	q_0_ n_SSB_(+)n_SSB_(S2) + q_1_/2 [n_DSB_(+)n_SSB_(S) + n_SSB_(++)n_SSB_(S) + n_SSB_(+)n_DSB_(S) + n_SSB_(+)n_SSB_(+)]

**Table 2:** Evaluation of *J_TOT_*(*ε*)*-J_D_* = 1 and 2 terms in multinomial DNA damage model for SSB, DSB, and BD. The constant factors, *p_A_*, *p_B_*, and *p_C,_* are suppressed in the formulas and are found easily by considering the definition of the term defined in the second column. The addition of probabilities is shown, while marginal distributions are selected for lesions of specific types.

Order	Term	Components
1	P_A_	n_SSB_(S)
1	P_B_	r_1_n_SSB_(S) + r_2_n_BD_(1)
1	P_C_	n_BD_(1)
2	P_A_P_A_	q_0_n_SSB_(S2) + q_1_n_DSB_(S) + q_1_n_SSB_(+)
2	P_A_P_B_	r_1_q_0_n_SSB_(S2) + r_1_q_1_n_DSB_(S) + r_1_q_1_n_SSB_(+) + r_2_n_SSB_(S)n_BD_(1)+r_3_n_SSB_(S)
2	P_A_P_C_	n_SSB_(S)n_BD_(1)
2	P_B_P_B_	r_1_^2^ [q_0_n_SSB_(S2) + q_1_n_DSB_(S) + q_1_n_SSB_(+)] + 2r_1_r_2_n_SSB_(S)n_BD_(1) + r_2_^2^n_BD_(2) + r_3_[r_1_n_SSB_(S) + r_2_n_BD_(1)]
2	P_B_P_C_	[r_1_n_SSB_(S) + r_3_]n_BD_(1) + r_2_n_BD_(2)
2	P_C_P_C_	n_BD_(2)

**Table 3:** Evaluation of *J_TOT_*(*ε*)-*J_D_* = 3 terms in multinomial DNA damage model for SSB, DSB, and BD. The constant factors, p_A_, p_B_, and p_C,_ are suppressed in the formulas and are found easily by considering the definition of the term defined in the left-hand column. The addition of probabilities is shown, while marginal distributions are selected for lesions of specific types.

Term	Components
P_A_P_A_P_A_	q_0_^2^n_SSB_(S3) + 2q_0_q_1_[n_DSB_(S)n_SSB_(S) + n_SSB_(+)n_SSB_(S)] + q_1_^2^[3n_DSB_(+) + n_SSB_(++)]
P_A_P_A_P_B_	r_1_{q_0_^2^ n_SSB_(S3) + q_0_q_1_[n_DSB_(S)n_SSB_(S) + n_SSB_(+)n_SSB_(S)]+ q_1_^2^/2[3n_DSB_(+) + n_SSB_(++)]} + r_2_{q_0_n_SSB_(S2) + q_1_[n_DSB_(S) + n_SSB_(+)]}n_BD_(1)+ r_3_{q_0_n_SSB_(S2) + q_1_[n_DSB_(S) + n_SSB_(+)]}
P_A_P_A_P_C_	A_2_ n_BD_(1)
P_A_P_B_P_B_	r_1_^2^{q_0_^2^n_SSB_(S3) + 3q_0_q_1_[n_SSB_(S)n_SSB_(S) + n_SSB_(+)n_SSB_(S)] + q_1_^2^/2[3n_DSB_(+) + n_SSB_(++)]} + 2r_1_r_2_{q_0_n_SSB_(2) + q_1_n_DSB_(S) + q_1_n_SSB_(+)]n_BD_(1) + r_2_r_3_n_SSB_(2)n_BD_(1)} + r_1_r_3_{q_0_n_SSB_(S3) + q_1_n_DSB_(S)n_SSB_(S) + q_1_n_SSB_(+)n_SSB_(S) + q_0_n_SSB_(S2) + q_1_n_DSB_(S) + q_1_n_SSB_(+)}+ r_2_^2^n_SSB_(S)n_BD_(1) + r_3_^2^n_SSB_(2)
P_A_P_C_P_C_	n_SSB_(S) n_BD_(2)
P_A_P_B_P_C_	r_1_[q_0_n_SSB_(S2) + q_1_n_DSB_(S) + q_1_n_SSB_(+)]n_BD_(1) + r_2_n_SSB_(S)n_BD_(3) + r_3_n_SSB_(S)n_BD_(1)
P_B_P_B_P_B_	~Β_2_ r_2_n_BD_(1) + r_3_B_1_
P_B_P_B_P_C_	B_2_ r_2_n_BD_ (1) + r_3_B_1_
P_B_P_C_P_C_	[r_1_n_SSB_(S) + r_3_]n_BD_(2) + r_2_n_BD_(3)
P_C_P_C_P_C_	n_BD_(3)

**Table 4:** Evaluation of *J_TOT_*-*J_D_* = 4 terms in multinomial DNA damage model for SSB and DSB. The constant factors, p_A_, p_B_, and p_C,_ are suppressed in the formulas and are found easily by considering the definition of the term defined in the left-hand column. The addition of probabilities is shown, while marginal distributions are selected for lesions of specific types.

Term	Components
P_A_P_A_P_A_P_A_	See Equation (17)
P_A_P_A_P_A_P_B_	r_1_A_4_ + [r_2_n_BD_(1) + r_3_]A_3_
P_A_P_A_P_A_P_C_	A_3_ n_BD_(1)
P_A_P_A_P_B_P_B_	See Equation (15)
P_A_P_A_P_C_P_C_	A_2_n_BD_(2)
P_A_P_A_P_B_P_C_	r_1_A_3_n_BD_(1) + [r_2_n_BD_(2) + r_3_n_BD_(1)]A_2_
P_A_P_B_P_B_P_C_	A_1_B_2_ n_BD_(1)
P_A_P_B_P_B_P_B_	~A_1_B_2_ r_2_n_BD_(1)
P_A_P_C_P_C_P_C_	n_SSB_(S)n_BD_(3)
P_A_P_B_P_C_P_C_	A_1_B_1_n_BD_(2)
P_B_P_B_P_B_P_B_	~B_2_ [r_2_^2^n_BD_(2) + r_3_^2^ + 2r_2_r_3_n_BD_(1)]
P_B_P_B_P_B_P_C_	r_1_^2^ [q_0_n_SSB_(S2) + q_1_n_DSB_(S) + q_1_n_SSB_(C)]n_BD_(2) + 2r_1_r_2_n_SSB_(S)n_BD_(3) + r_2_^2^n_BD_(4)
P_B_P_B_P_C_P_C_	B_2_n_BD_(2)
P_C_P_C_P_C_P_A_	n_BD_(3)n_SSB_(S)
P_C_P_C_P_C_P_B_	[r_1_n_SSB_(S) + r_3_]n_BD_(3) + r_2_n_BD_(4)
P_C_P_C_P_C_P_C_	n_BD_(4)

**Table 5:** Predictions of yields of several types of DNA lesions per Gbp per Gy for several types of ionizing radiation.

Radiation Type/ Lesion	Electrons (100 keV)	^4^He (1 MeV/u, LET = 104 keV/μm)	^12^C (10 MeV/u, LET = 163 keV/μm)	^12^C (1000 MeV/u, LET = 8 keV/μm)
SSB-S	26.5	10.1	14.8	20.7
SSBS2	6.1	0.7	0.9	1.3
SSB+	6.1	3.1	4.0	5.4
SSB++	0.5	0.3	0.3	0.4
Total SSB *	63.4	18.5	25.7	35.4
DSB-S	6.5	3.3	4.3	5.8
DSB+	0.8	0.7	0.7	0.8
DSB++	2.7	5.3	4.3	2.8
Total DSB	9.9	9.3	9.3	9.4
BD-1	36.7	15.0	20.5	27.0
BD-2	17.2	10.9	12.7	15.4
BD-3	8.7	8.0	8.1	8.4
BD > 3	4.4	7.5	6.2	4.4
Total BD *	112.9	90.7	95.3	100.6

* Values weighted by number of SSBs or BD in a lesion.

## Data Availability

Request can be made to the author.
